# Contrast-Permeated Suture Tape in Orthopedic Practice: Imaging Characteristics and Mechanical Performance

**DOI:** 10.7759/cureus.110181

**Published:** 2026-06-03

**Authors:** Jordan D Shearer, Alexander C Chong, Todd Pottinger, Bruce E Piatt

**Affiliations:** 1 Sanford Medical Education, Sanford Health, Fargo, USA; 2 Orthopedic Surgery, University of Minnesota Medical School, Minneapolis, USA

**Keywords:** biomechanical phenomena, contrast media, fluoroscopy, iohexol, sutures, suture tape

## Abstract

Suture tapes are widely used in orthopedic procedures, providing superior tissue repair through higher tensile strength than traditional round sutures. Their broad, flat profile makes them especially well-suited for cerclage fixation, distributing pressure over a larger surface area and enhancing overall stability. Cerclage fixation remains a widely utilized technique in orthopedic practice, serving to reinforce structurally compromised tissue through circumferential mechanical support. Suture-based cerclage fixation has emerged as an alternative to traditional metal wires or cables for the management of periprosthetic fractures. However, a key limitation of suture-based cerclage fixation is its limited visibility on standard imaging modalities, which restricts both intraoperative verification and postoperative assessment. This technical report aimed to (1) report the feasibility of incorporating a commercially available iodine-based contrast medium into suture tape to render it radiopaque under fluoroscopy, and (2) assess the impact of this modification on the suture tape’s key mechanical properties.

Contrast-permeated suture tape was prepared using a standardized protocol to ensure reproducibility, consisting of one-minute immersion in an iodine-containing contrast medium, gentle manual wringing between two fingers to remove excess contrast, and approximately two minutes of air-drying under ambient laboratory conditions prior to testing. Standardized fluoroscopy was performed under identical exposure settings. Radiographic visibility of contrast-permeated (n = 4) and non-contrast-permeated (n = 4) suture tapes was assessed using a cadaveric model simulating an oblique mid-shaft humeral fracture under three applied tension conditions (no tension, 50% of maximum tension, and 75% of maximum tension). Visibility was assessed using a standardized ordinal grading scale. Mechanical properties of the suture tape in both groups (n = 12 per group) were assessed using dynamic creep and ultimate load-to-failure testing.

Contrast-permeated suture tape was radiopaque under fluoroscopy across all tension levels, whereas non-contrast-permeated tape remained radiolucent. Dynamic creep displacement was 2.3 ± 0.6 mm for contrast-permeated suture tape and 2.1 ± 0.7 mm for non-contrast tape, with no statistically significant difference (p = 0.46). Ultimate load-to-failure was significantly higher for contrast-permeated tape (737 ± 46 N) compared with non-contrast tape (642 ± 59 N, p < 0.05). One possible explanation for the higher ultimate load-to-failure observed in the contrast-permeated group is that air exposure produced an adhesive effect in the iodine-based contrast medium, increasing inter-strand friction and reducing knot slippage, thereby enhancing ultimate failure strength.

In conclusion, iodine-based contrast-permeated suture tape is clinically feasible and enhances fluoroscopic visibility without compromising mechanical performance. It offers a practical alternative to conventional sutures or suture tape constructs where clear intraoperative and postoperative visualization is essential.

## Introduction

Most suture tapes are flat-braided and composed of non-absorbable ultra-high molecular weight polyethylene (UHMWPE) combined with polyester. Widely used in orthopedic procedures, they provide superior tissue repair by offering higher tensile strength than traditional round sutures. Their broad, flat profile makes them especially well-suited for cerclage fixation, as it distributes pressure over a larger surface area and enhances overall stability.

Cerclage fixation remains a widely utilized technique in orthopedic practice, serving to reinforce structurally compromised tissue through circumferential mechanical support. Despite its widespread use, optimal material selection for cerclage has not been definitively established. In recent years, suture-based cerclage fixation has emerged as an alternative to traditional metal wires or cables for the management of periprosthetic fractures [1-5]. However, a key limitation of currently available suture and suture tape constructs is their radiolucency, which limits visualization on standard imaging modalities. Consequently, intraoperative assessment of suture placement and construct integrity may be challenging, while postoperative imaging may not reliably distinguish the suture construct from surrounding soft tissue. This limited radiographic visibility can complicate confirmation of implant positioning, evaluation of construct failure, and assessment during revision procedures.

The development of iodine-based contrast-permeated suture tape represents an effort to address this limitation by integrating radiopacity into a mechanically functional implant. Iodinated contrast agents, such as iohexol, are water-soluble intravenous radiocontrast media that enhance the visibility of anatomical structures during radiographic procedures [6]. Iohexol has a well-documented safety profile and is among the most used contrast agents in computed tomography (CT) scans [7-10]. Incorporating such agents into suture materials offers the potential to improve visualization under fluoroscopy and other imaging modalities.

However, several critical questions remain. First, how can iodinated contrast agents be effectively incorporated into suture tape? Second, does contrast-permeated suture cerclage tape demonstrate improved radiographic visibility and clinical detectability? Third, does the incorporation of an iodine-based contrast medium compromise the mechanical integrity of the suture tape? Accordingly, this technical report aimed (1) to report the feasibility of incorporating an iodine-based contrast medium into suture tape to render it radiopaque under fluoroscopy, and (2) to assess the impact of this modification on key mechanical properties of the suture tape.

## Technical report

Contrast-permeated suture tape preparation

Commercially available suture tape (FiberTape® Cerclage, Arthrex, Naples, FL) was used as the control material. The contrast-permeated suture tape (experimental group) was prepared using the same suture tape, permeated with an iodine-based contrast medium (Omnipaque^TM^ 350 (iohexol), GE Healthcare, Oslo, Norway). To ensure reproducible and standardized contrast permeation across all specimens, all suture tape samples were prepared using an identical protocol. Each suture tape specimen was immersed in the contrast medium for one minute to facilitate uniform contrast uptake. Following immersion, excess contrast medium was removed using consistent wringing with gentle manual compression between two fingers to minimize surface pooling while preserving intrafibrillar permeation. The suture tape was then air-dried under ambient laboratory conditions for approximately two minutes prior to testing.

Bench-top radiographic visibility assessment under fluoroscopy

**Figure 1:**
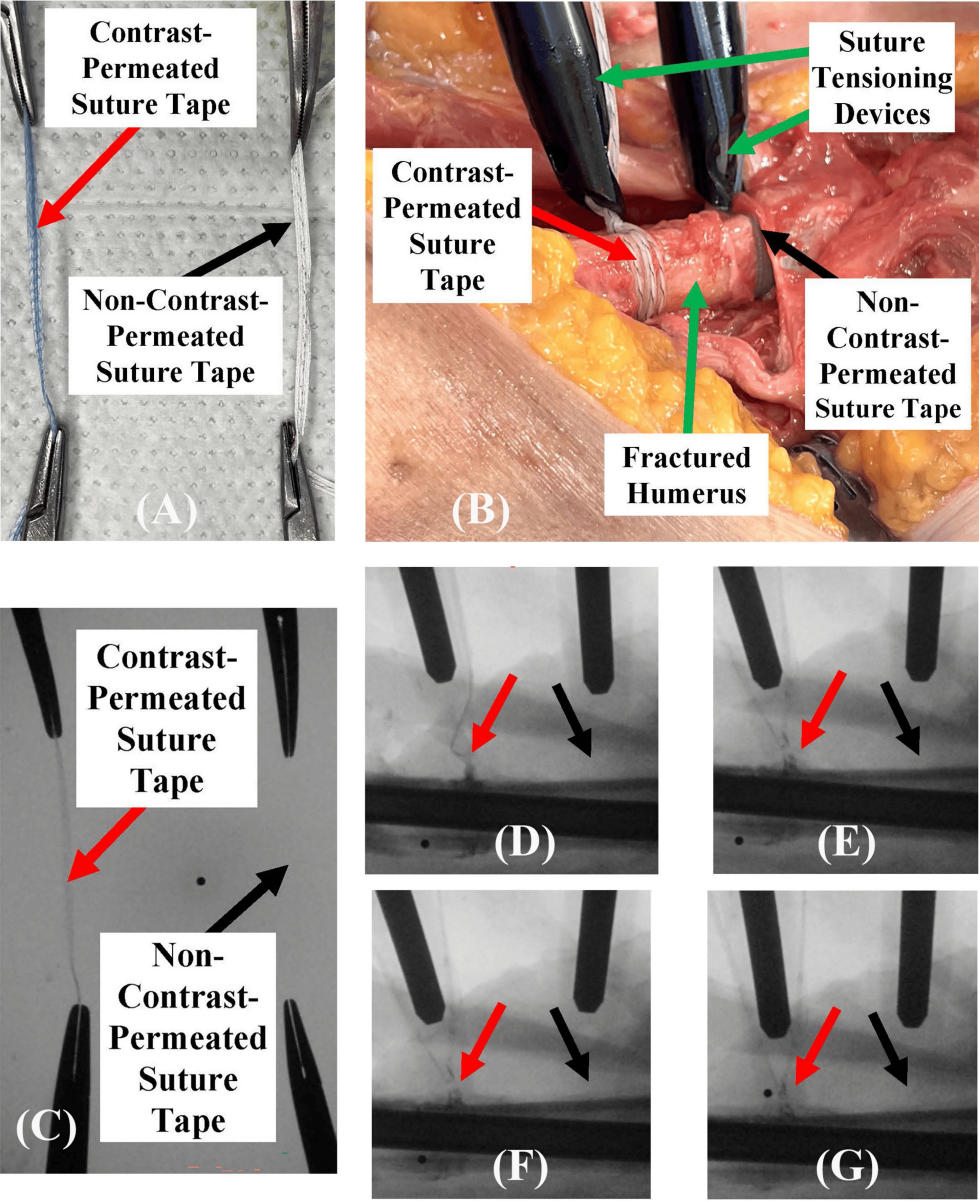
Bench-top and cadaveric radiographic assessment with contrast-permeated suture tape (red arrow) alongside non-contrast-permeated suture tape (black arrow). (A) Bench-top radiographic visibility assessment experimental setup. (B) Cadaveric radiographic visibility assessment experimental setup. (C) Radiographic image illustrating bench-top radiographic visibility assessment result. (D) Illustration of the cadaveric radiographic visibility assessment without tension applied, fluoroscopic image result. (E) Illustration of the cadaveric radiographic visibility assessment with 50% maximum tension applied. (F) Illustration of the cadaveric radiographic visibility assessment with 75% maximum tension applied. (G) Illustration of the cadaveric radiographic visibility assessment after a five-minute holding period at 75% maximum tension.

Radiographic visibility was assessed using standard fluoroscopic imaging. Suture samples from both groups were placed on a radiolucent platform, with each end secured using a hemostat to facilitate visualization (Figure 1A). Radiographic visibility was independently evaluated by two orthopedic surgeons using a standardized ordinal grading scale (0 = not visible; 1 = visible).

Cadaveric radiographic visibility assessment under fluoroscopy

For the cadaveric radiographic visibility assessment, a fresh-frozen, non-preserved humerus was obtained from an institutionally approved tissue supplier and thawed to room temperature prior to preparation. A simulated oblique mid-shaft humeral fracture was created using a surgical saw, and an intramedullary device was inserted across the fracture site after canal preparation to replicate humeral fracture fixation. Two cerclage fixation constructs were then applied: one using contrast-permeated suture cerclage tape and the other using non-contrast-permeated suture cerclage tape (n = 4 per group). In both groups, the suture tape was looped circumferentially around the fractured humerus at multiple levels. The suture tails were then passed through a lasso-like loop to create a self-locking construct. Techniques were performed to closely replicate standard in vivo reduction and fixation (Figure 1B), followed by simulated wound closure prior to radiographic imaging.

Radiographic visibility of the suture cerclage constructs in both groups was evaluated under three load conditions using a suture tensioning device (FiberTape® Cerclage disposable tensioner, Arthrex, Naples, FL): (1) no tension; (2) 50% of maximum tension (corresponding to the second gauge marking on the tensioning device); and (3) 75% of maximum tension (third gauge marking). Anteroposterior fluoroscopic images of the humeral midshaft were obtained at each tension level. An additional radiograph was acquired after maintaining 75% of maximum tension for five minutes in both groups to assess changes in visualization under sustained load. All fluoroscopic imaging was performed using standardized exposure parameters to minimize variability in radiographic appearance between specimens and testing conditions. Radiographic images were evaluated using a dedicated picture archiving and communication system (PACS) (Sectra IDS7, Sectra AB, Linköping, Sweden) and a standardized ordinal grading scale (0 = not visible; 1 = visible).

Mechanical property assessment of suture tape

**Figure 2:**
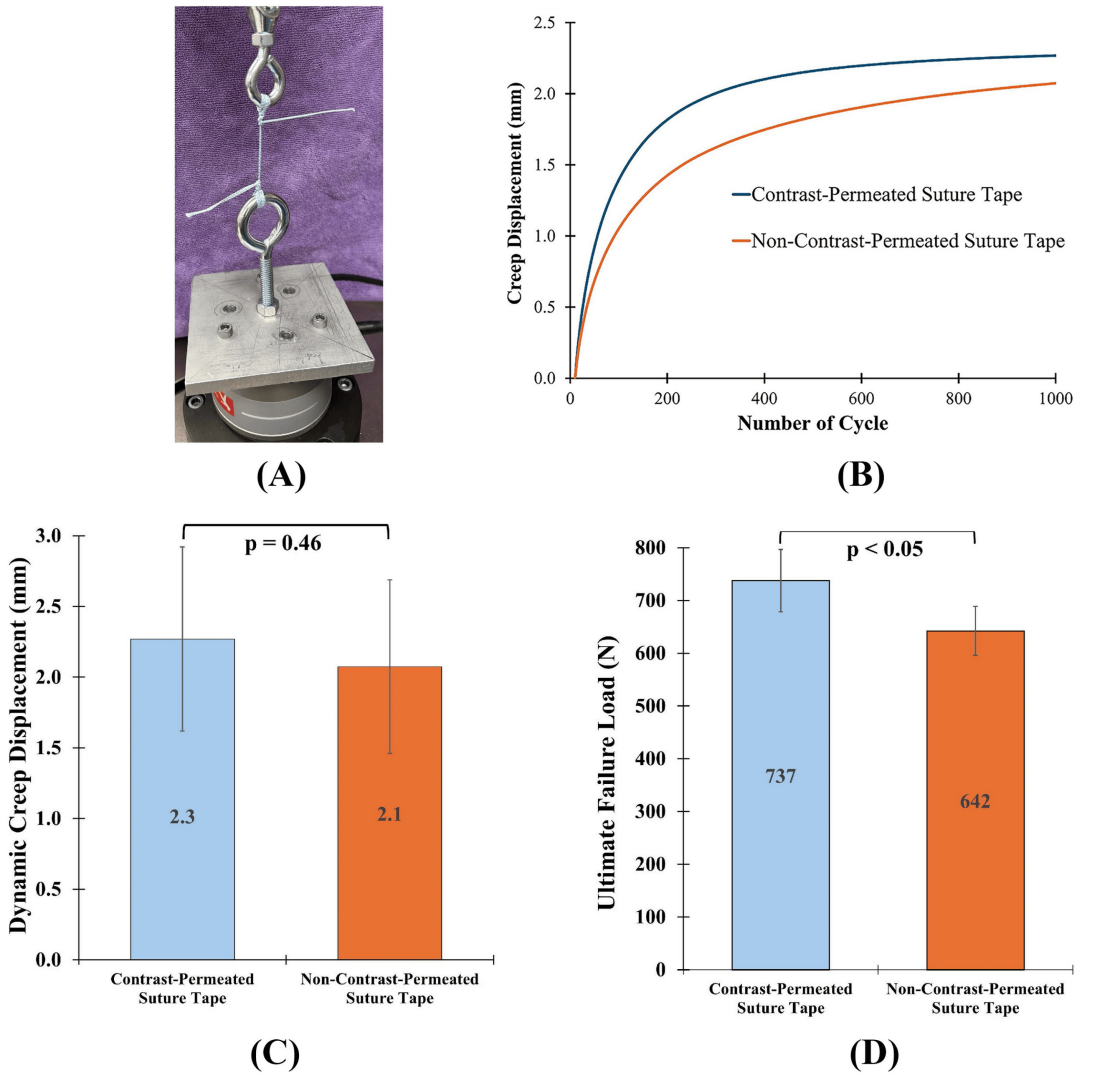
Evaluation of mechanical properties for contrast-permeated and non-contrast-permeated suture tape. (A) Experimental setup. (B) Creep displacement with cyclic loading test results. (C) Dynamic creep displacement test results. (D) Ultimate load-to-failure test results.

All mechanical testing was performed using a servo-hydraulic materials testing system (Model 8874; Instron, Norwood, MA). A total of 24 samples were evaluated, including 12 contrast-permeated suture tapes and 12 non-contrast-permeated controls. Each suture tape sample was secured around two round hooks attached to the actuator and load cell of the testing system and tied using five half-hitch knots to minimize slippage and ensure consistent loading conditions (Figure 2A).

Dynamic creep testing

Each sample was preloaded to 10 N and standardized to an initial gauge length of 50 mm. This preload was applied to eliminate slack and initial settling in the suture material, thereby establishing a consistent baseline for testing. Samples were then subjected to cyclic tensile loading between 10 N and 100 N at a frequency of 0.5 Hz for 1,000 cycles, consistent with previously published protocols [11,12]. Load and displacement data were recorded at a sampling rate of 100 Hz, with creep displacement captured at five-cycle intervals. Dynamic creep displacement was defined as the increase in peak displacement between the 10th and 1,000th loading cycles.

Load-to-failure testing

Each sample was preloaded to 10 N and then loaded to failure at a constant crosshead displacement rate of 1 mm/second. Load and displacement were continuously recorded at a sampling rate of 100 Hz. Ultimate failure load was defined as the maximum load sustained prior to structural failure.

Results

In both bench-top and cadaveric radiographic visibility assessments, contrast-permeated suture tape appeared visible under fluoroscopy across all tested conditions, whereas non-contrast-permeated suture tape remained radiolucent regardless of the applied tension (Figures 1C-1G). The radiographic images also indicated that radiopacity did not appear to be affected by increasing tension or prolonged loading conditions.

Data normality for the creep displacement and ultimate failure strength was assessed using the Shapiro-Wilk and Kolmogorov-Smirnov tests, which confirmed that both datasets were normally distributed (p > 0.05).

For dynamic creep evaluation, creep displacement during cyclic loading showed a difference between the two groups in the early loading phase, with divergence persisting through approximately the 200^th^ cycle (Figure 2B). The contrast-permeated suture tape demonstrated a mean creep displacement of 2.3 ± 0.6 mm (range: 1.5-3.3 mm), compared with 2.1 ± 0.7 mm (range: 1.1-3.3 mm) in the non-contrast-permeated group (Figure 2C). However, independent Student’s t-test analysis indicated that this difference was not statistically significant (p = 0.46).

Comparison of ultimate load-to-failure demonstrated that contrast-permeated suture tapes exhibited a higher mean ultimate failure strength (737 ± 46 N) than non-contrast-permeated suture tapes (642 ± 59 N) (Figure 2D). Independent Student’s t-test analysis indicated that this difference was statistically significant (p < 0.05).

## Discussion

Cerclage fixation is frequently used for osteotomies and periprosthetic fractures when screw or plate fixation is limited by inadequate bone purchase. Suture tape cerclage devices are designed to be less irritating and easier to handle than traditional metallic cerclage devices. However, their radiolucent nature can be a limitation in fracture care, where precise placement is critical; improper positioning may compromise fixation biomechanics or contribute to complications such as nerve impingement [13,14].

The present technical report demonstrates that a commercially available suture tape can be effectively modified into a contrast-permeated cerclage construct suitable for orthopedic applications. The findings show that contrast-permeated suture cerclage tape exhibits radiopacity on intraoperative fluoroscopy, whereas non-contrast-permeated tape remains radiolucent under all tested conditions. A radiopaque suture may facilitate intraoperative confirmation of cerclage placement, particularly in anatomically complex or delicate regions such as periprosthetic fracture sites, where accurate cerclage placement and postoperative monitoring are essential. The concept of radiopaque sutures is not entirely novel, as prior studies have demonstrated their clinical utility [15,16]. Nevertheless, a contrast-permeated cerclage construct may enable more accurate postoperative assessment, addressing a key limitation of conventional suture materials.

The present report also demonstrates that incorporating the iodine-based contrast does not adversely affect the mechanical performance of the suture tape. Notably, contrast-impregnated suture tapes demonstrated significantly higher ultimate load-to-failure compared to non-contrast-impregnated controls. One potential explanation for this finding is the possible adhesive effect of the iodine-based contrast medium following air exposure. This effect may have enhanced knot security by increasing inter-strand friction and reducing knot slippage. From a biomechanical perspective, contrast-permeated suture tape exhibits dynamic creep and ultimate failure load comparable to, or greater than, non-contrast-permeated controls, indicating that the addition of contrast medium does not compromise its structural integrity.

**Figure 3:**
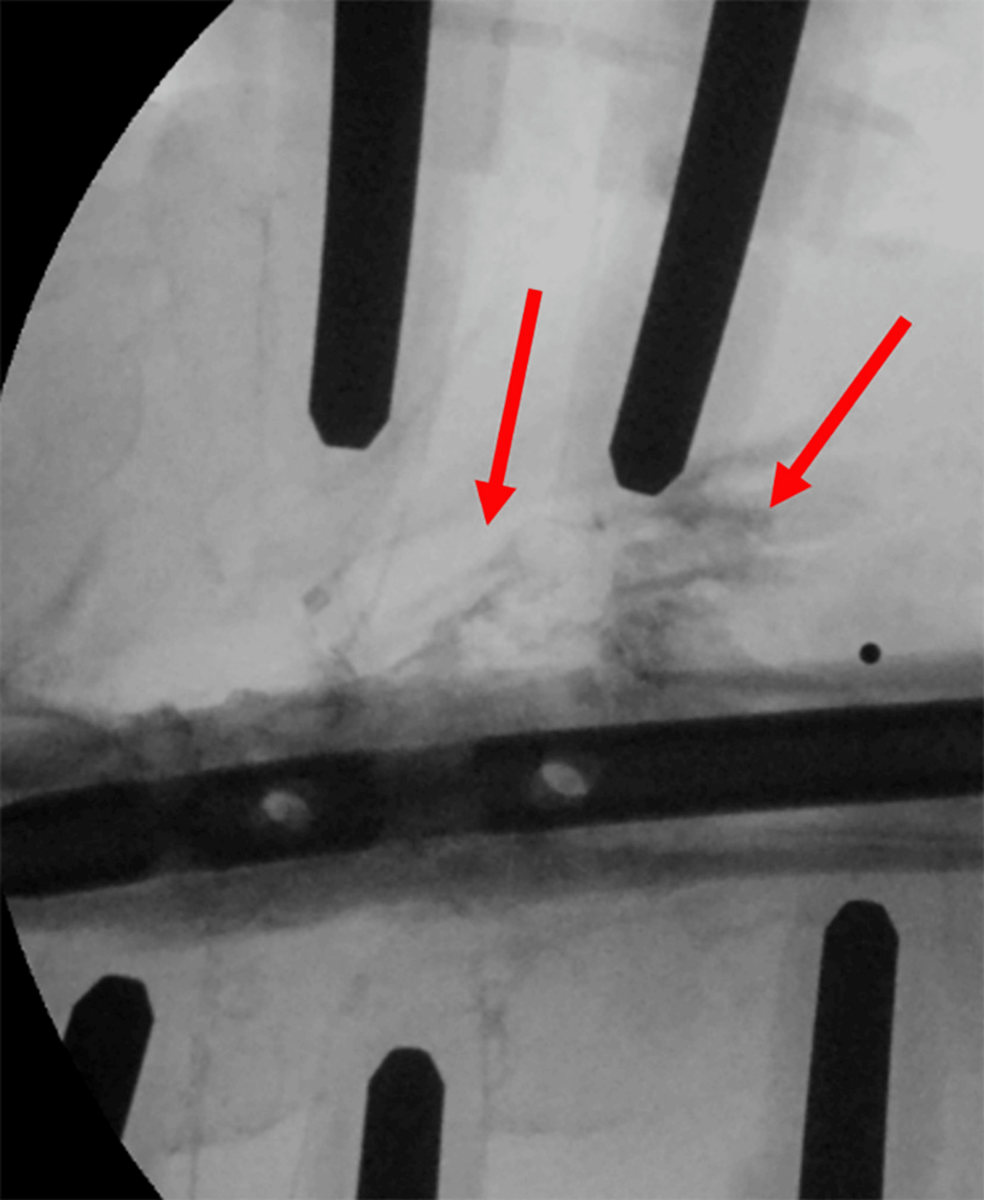
Potential pitfall of excess contrast without careful application technique.

Notably, the findings of this study suggest that contrast-permeated suture cerclage tape should be applied with particular care. Substantial movement of the contrast-permeated suture tape was observed to potentially obscure visualization of fracture reduction or cerclage positioning under fluoroscopy (Figure 3), which may limit its effectiveness as an imaging adjunct in dynamic intraoperative settings.

This technical report has several limitations that should be acknowledged. First, a single cadaveric model was used for radiographic visibility assessment, which may not fully replicate in vivo conditions. In particular, the absence of a functioning circulatory system may have affected the absorption, distribution, and clearance of the contrast medium compared with living tissue. Second, although iohexol has a well-established safety profile, the potential long-term effects of contrast incorporation on tissue biocompatibility, suture durability, the sterilization process, and in vivo washout or degradation of the permeated contrast medium were not evaluated. Third, cerclage construct configurations were not systematically investigated. Fourth, mechanical testing of the suture tape was limited to a controlled laboratory environment and to dynamic creep and single load-to-failure assessments, which do not fully reflect the complex, multifactorial loading conditions encountered clinically. Fifth, knot-tying techniques on cerclage constructs were not systematically investigated, as these factors were beyond the scope of the present study. Further investigation, including animal models and prospective clinical trials with longer follow-up, is needed to validate these findings. Additional imaging assessments using computed tomography (CT) and magnetic resonance imaging (MRI) may also help define the clinical utility and translational potential of contrast-permeated cerclage tape in surgical practice.

## Conclusions

In conclusion, iodine-based contrast-permeated suture tape is clinically feasible and enhances fluoroscopic visibility without compromising mechanical performance. It may serve as a viable alternative to conventional sutures or suture tape constructs, particularly in procedures where clear intraoperative and postoperative visualization is essential, and ease of handling is beneficial. However, clinicians should exercise caution when using contrast-permeated suture tape, as the long-term in vivo biocompatibility and tissue response associated with contrast-permeated suture materials have not yet been established, and in vivo performance may differ from the present experimental findings.
